# Phakomatosis Pigmentovascularis (Type IIb) With Bilateral Glaucoma and Urrets‐Zavalia Syndrome Following MicroPulse TSCPC: A Case Report

**DOI:** 10.1155/crop/3631637

**Published:** 2026-05-03

**Authors:** Ali Alshehri, Yasser Bin Thabet, Mohammed Naji Almutairi

**Affiliations:** ^1^ Ophthalmology Department, Glaucoma Department, Prince Sultan Military Medical City, Riyadh, Saudi Arabia, psmmc.med.sa; ^2^ College of Medicine, King Saud bin Abdulaziz University for Health Sciences, Riyadh, Saudi Arabia, ksau-hs.edu.sa

**Keywords:** oculodermal melanocytosis, phakomatosis pigmentovascularis, Urrets-Zavalia syndrome, UZS

## Abstract

**Purpose:**

We are aimed at describing the clinical course and management of a rare case of phakomatosis pigmentovascularis (PPV Type IIb) presenting with bilateral glaucoma, complicated by bilateral Urrets‐Zavalia syndrome (UZS) following MicroPulse transscleral cyclophotocoagulation (MP‐TSCPC).

**Observations:**

A 6‐year‐old girl with PPV Type IIb and syndromic glaucoma underwent MP‐TSCPC as an initial surgical intervention for uncontrolled intraocular pressure (IOP). Postoperatively, the patient developed bilateral fixed dilated pupils consistent with UZS. Due to persistent IOP elevation and the complication of UZS, bilateral deep sclerectomy (DS) was performed, resulting in adequate IOP control. (10‐mmHg OD, 17‐mmHg OS) and gradual improvement in pupillary function. No further complications were observed. The case was classified under PPV Type II based on the coexistence of vascular and pigmentary anomalies. A review of the literature confirms the rarity of such a constellation of findings and the sparsity of reported bilateral UZS following MP‐TSCPC.

**Conclusion and Importance:**

This case highlights that PPV Type IIb requires careful examination because of the risk of associated glaucoma. UZS should be considered as a possible complication after MP‐TSCPC, especially when both eyes are treated. In complex cases with vascular involvement, DS can provide effective and safe IOP control.

## 1. Introduction

Phakomatosis pigmentovascularis (PPV) is an uncommon condition characterized primarily with a vascular malformation, typically a capillary malformation, and dermal melanosis such as a nevus of Ota or Mongolian spot, with or without systemic extracutaneous involvement. [1] This rare association has mostly been reported in Asian populations and in dermatological literature, and due to its rarity, the exact prevalence remains unknown. [2]

PPV is considered a mosaic abnormality of vasomotor nerves and melanocytes, which leads to its distinctive skin manifestations. There are five recognized types of PPV, based on the characteristics of the vascular and pigmentary malformations. Each type is further divided into subtypes A and B, depending on whether there is systemic involvement (which is observed in subtype B). Type II, featuring a port wine stain combined with dermal melanosis, is the most observed form. [3, 4] The full classification of PPV is summarized in Table 1. Based on Happle′s revised classification, our patient fits the diagnostic criteria for PPV Type IIb, due to the coexistence of capillary malformation, dermal melanocytosis, and systemic involvement, including bilateral congenital glaucoma. [5] Reports of PPV Type IIb with bilateral glaucoma remain limited in the literature. This case illustrates the diagnostic and management challenges associated with this rare neurocutaneous condition and the need for individualized surgical planning in complex pediatric glaucoma.

**Table 1:** Classification of phakomatosis pigmentovascularis (PPV) based on clinical features, incorporating Happle′s revised nomenclature.

Type	Clinical features
I	Capillary malformation, epidermal nevus
II (Cesioflammea)	Capillary malformation, dermal melanosis (lumbosacral dermal melanocytosis, nevus of Ota)
III (Spilorosea)	Capillary malformation, nevus spilus, nevus anaemicus
IV (Unclassified)	Capillary malformation, dermal melanosis (lumbosacral dermal melanocytosis, nevus of Ota), nevus spilus, nevus anaemicus
V (Cesiomarmorata)	Cutis marmorata telangiectatica congenita, dermal melanosis (lumbosacral dermal melanocytosis, nevus of Ota)

## 2. Methods

This is a descriptive case report of a pediatric patient with PPV Type IIb and bilateral congenital glaucoma, evaluated at Prince Sultan Military Medical City (PSMMC), Riyadh, Saudi Arabia. Ethics approval was obtained from the Institutional Review Board of PSMMC, and written informed consent was obtained from the patient′s parents for publication of clinical data and images.

The patient underwent comprehensive ophthalmic evaluation, including intraocular pressure (IOP) measurement, anterior segment and fundus examination, A‐scan biometry, and central corneal thickness (CCT) measurement. All values were reported using standard units (IOP in mmHg, axial length in mm, CCT in *μ*m).

## 3. Case Report

**Figure 1:**
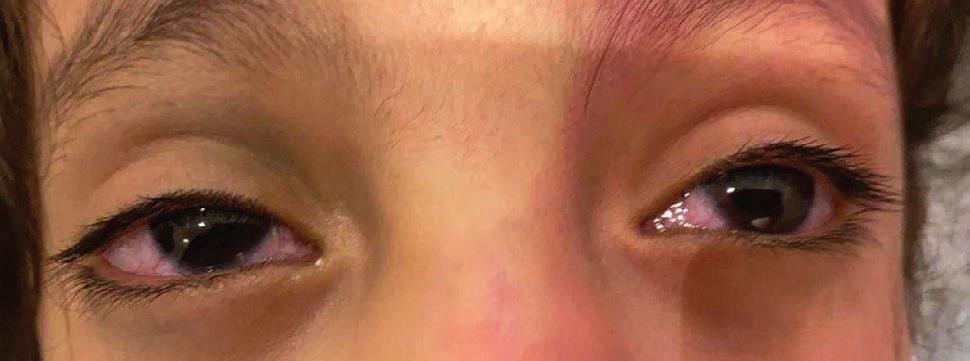
Bilateral episcleral vascular engorgement with prominent dilated episcleral vessels in both eyes. A facial port wine stain is also visible on the forehead.

**Figure 2:**
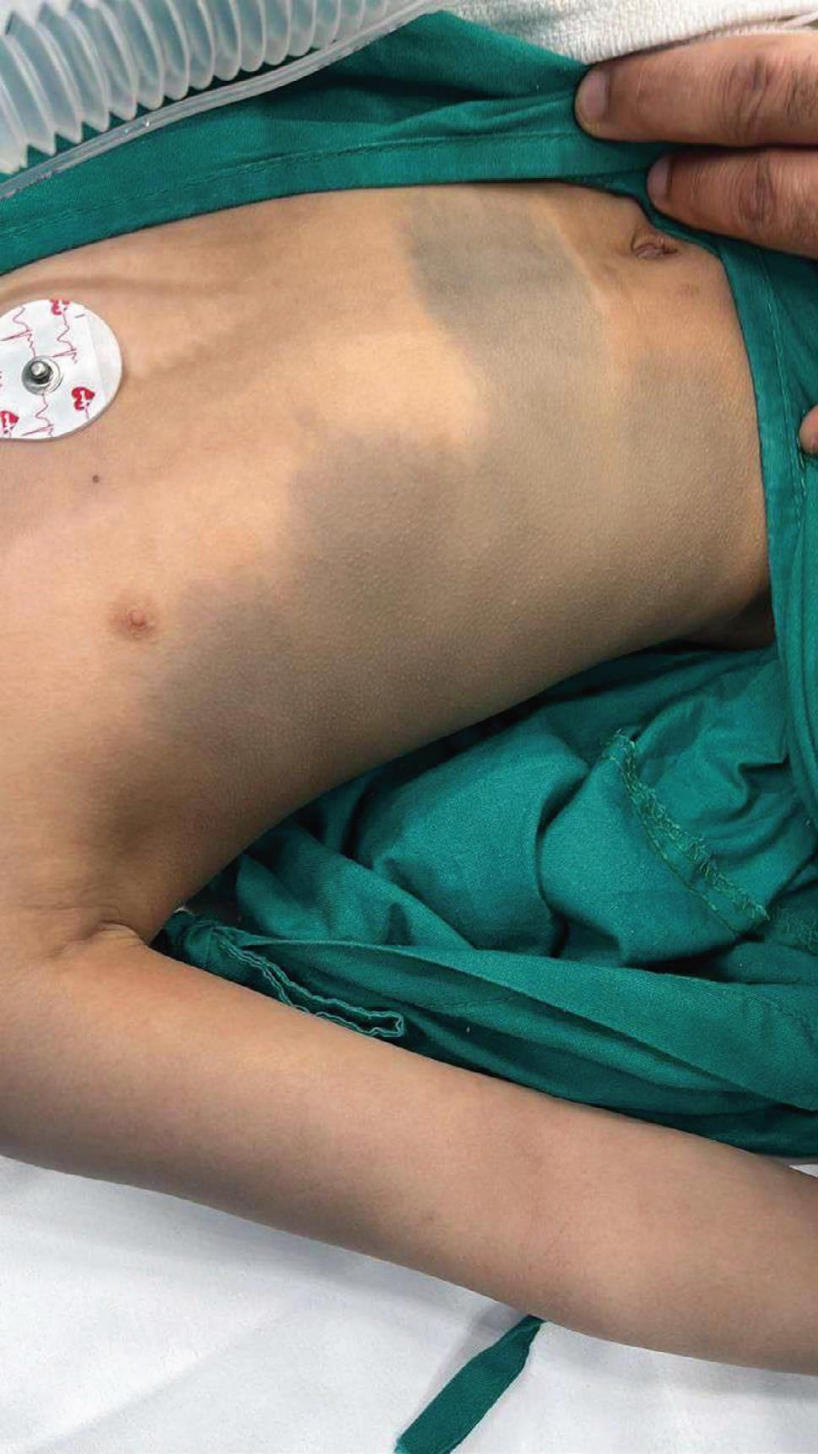
Cutaneous vascular malformation over the thoracoabdominal region demonstrating a large bluish patch on the right flank and lower chest.

A 6‐year‐old girl, known case of bilateral congenital glaucoma secondary to SWS, presented for further evaluation. General examination revealed a left‐sided facial port wine stain involving the upper eyelid, and ipsilateral grayish conjunctival pigmentation consistent with nevus of Ota (Figure 1). Dermatological examination confirmed bluish gray dermal melanocytosis (Mongolian spots) over the trunk and flank (Figure 2). All skin lesions were present since birth. Although the patient was initially followed as a case of congenital glaucoma associated with SWS, the coexistence of capillary malformation, dermal melanocytosis (nevus of Ota), and systemic involvement supports a final diagnosis of PPV Type IIb. There were no neurological deficits on clinical examination; the patient had previously undergone neuroimaging at another medical center as part of the diagnostic evaluation and continued regular follow‐up there. Therefore, repeat neuroimaging was not performed at our institution, to avoid unnecessary radiation exposure and in consideration of the risk benefit profile. Best visual behavior was central, steady, and maintained fixation in both eyes. IOP was 36‐mmHg OD and 26‐mmHg OS (iCare tonometry). Axial lengths were 24.33‐mm OD and 24.14‐mm OS; CCT was 563‐*μ*m OD and 552‐*μ*m OS. Pupils were round, regular, and reactive to both light and near stimuli in both eyes at baseline.

The anterior segment was quiet aside from conjunctival pigmentation. Gonioscopy revealed Shaffer Grade 3 open angles in all quadrants. Fundoscopy revealed a choroidal hemangioma in the left eye and cup‐to‐disc ratios of 0.7 in both eyes. Initial management included full topical antiglaucoma medications and oral acetazolamide. Despite treatment, IOP remained elevated after 2 weeks. Given the increased risk of suprachoroidal hemorrhage and intraoperative complications in eyes with associated choroidal hemangioma, a minimally invasive cyclodestructive approach was selected initially. MP‐TSCPC has been reported to offer controlled energy delivery with reduced collateral tissue damage, making it a reasonable first surgical option in complex pediatric glaucoma cases. MP‐TSCPC was performed 2 weeks after initial medical therapy due to persistently elevated IOP and it was performed in accordance with IRIDEX Cyclo G6 protocol, using a MicroPulse P3 probe at 2000‐mW power, 31.3% duty cycle, and 90 s per hemisphere (total of 180 s per eye), based on the patient′s IOP range (26–36 mmHg).

On postoperative Day 1, IOP was 30 mmHg in both eyes. At 1 week, IOP remained elevated at 25‐mmHg OD and 24‐mmHg OS, and topical therapy with Xalatan and Azarga was initiated. At 1‐month visit, she developed fixed and dilated pupils measuring approximately 6‐mm OD and 5‐mm OS, with no reaction to light or near accommodation, suggestive of Urrets‐Zavalia syndrome (UZS). No signs of iris atrophy, posterior synechiae, or lens changes were observed during postoperative follow‐up. She subsequently underwent deep sclerectomy (DS) in both eyes. At Day 1 post‐op, IOP was 5‐mmHg OD and 6‐mmHg OS. At 1 month, IOP stabilized at 10‐mmHg OD and 17‐mmHg OS. The patient had a spontaneous partial recovery of the fixed, dilated pupils in both eyes, and the overall pupil size was smaller than previous visits. No postoperative complications were noted.

## 4. Discussion

In our case, initial management with MP‐TSCPC was selected as a minimally invasive, tissue sparing approach, given the presence of diffuse choroidal hemangioma and the potential risk of hemorrhagic complications with more invasive filtering procedures. MP‐TSCPC delivers repetitive short bursts of thermal energy designed to reduce collateral tissue damage compared with continuous wave cyclophotocoagulation. Although MP‐TSCPC is increasingly considered a safer alternative, particularly due to its reduced risk of hypotony and structural damage, its role in pediatric syndromic glaucoma remains incompletely defined. In complex eyes with associated vascular abnormalities, such as in PPV Type IIb, the theoretical safety advantage must be balanced against the possibility of unpredictable inflammatory or ischemic responses. This case illustrates that although MP‐TSCPC may serve as a less invasive initial intervention, clinicians should remain vigilant for rare but significant complications, especially when treating both eyes in a single session.

The development of bilateral fixed dilated pupils in our patient was consistent with UZS, a rare but recognized complication following anterior segment surgery and laser procedures. The pathogenesis of UZS remains incompletely understood. Proposed mechanisms include iris sphincter ischemia secondary to transient postoperative IOP elevation, direct thermal injury to the sphincter muscle, and disruption of parasympathetic innervation through ciliary nerve involvement. Although micropulse delivery reduces continuous energy exposure, cumulative thermal load may still induce reversible ischemic or neurogenic dysfunction. Bilateral simultaneous treatment may increase total thermal exposure, particularly in pediatric eyes that may be more susceptible to inflammatory or ischemic responses. [6, 7]

A recent case by Maneea et al. reported the first documented instance of bilateral UZS following MP‐TSCPC, with spontaneous partial recovery over 1.5 years [8] Their report highlighted similar clinical features to our patient, including middilated pupils unresponsive to light and accommodation. This underscores the importance of recognizing UZS as a potential complication of MP‐TSCPC, particularly in complex glaucoma cases with underlying ocular and systemic anomalies. Given inadequate IOP control following MP‐TSCPC and the appearance of fixed dilated pupils, we proceeded with DS in both eyes. DS, a nonpenetrating glaucoma procedure, is especially valuable in SWS due to its reduced risk of hypotony and choroidal complications. According to Almobarak et al., DS achieved significant and sustained IOP reduction in patients with SWS associated glaucoma, with a favorable safety profile [9]. Similarly, in our case, DS resulted in effective pressure control (IOP: 10‐mmHg OD, 17‐mmHg OS) and gradual resolution of the pupillary abnormalities, with no postoperative complications.

This case reinforces the role of DS as both an effective therapeutic and potentially rehabilitative option following MP‐TSCPC induced complications. Several prior reports have highlighted the clinical overlap of PPV, SWS, nevus of Ota, and congenital glaucoma, underscoring the importance of early recognition and comprehensive evaluation. Yang et al. described three pediatric patients with bilateral SWS, Ota nevus, and congenital glaucoma, two of whom had ocular and dermal findings consistent with PPV Type IIb and one with PPV type. [1] Recupero et al. reported an additional case involving a teenager with SWS and Ota nevus, emphasizing the rarity of this pigmentovascular. [10] Similarly, van der Merwe et al. detailed a young African patient with PPV Type II and associated glaucoma, reinforcing the value of early ophthalmic screening in patients with combined vascular and pigmentary. [11] It also contributes to the limited literature documenting bilateral UZS and its spontaneous improvement, emphasizing the need for vigilance and a tailored surgical approach in pediatric and syndromic glaucomas.

This case underscores the importance of individualized surgical planning in PPV Type IIb associated glaucoma and highlights the need for vigilance regarding rare but potentially reversible complications such as bilateral UZS.

## Author Contributions

**Ali Alshehri:** writing – original draft. **Yasser Bin Thabet:** writing – original draft, investigation. **Mohammed Naji Almutairi:** writing – review and editing.

## Funding

No funding was received for this manuscript.

## Conflicts of Interest

The authors declare no conflicts of interest.

## Data Availability

The data that support the findings of this study are available on request from the corresponding author. The data are not publicly available due to privacy or ethical restrictions.
